# Long Non-Coding RNA MALAT1 Contributed to the Proliferation of PNH Clone in Paroxysmal Nocturnal Hemoglobinuria Patients

**DOI:** 10.4274/tjh.galenos.2021.2021.0065

**Published:** 2021-08-25

**Authors:** Honglei Wang, Yingying Chen, Hui Liu, Zhaoyun Liu, Rong Fu

**Affiliations:** 1 Tianjin Medical University General Hospital, Department of Hematology, Tianjin, China

**Keywords:** Paroxysmal nocturnal hemoglobinuria, LncRNA, Clone proliferation, MALAT1

## To the Editor,

Paroxysmal nocturnal hemoglobinuria (PNH) is an acquired clonal disorder of hematopoietic stem cells caused by somatic mutation of the phosphatidylinositol glycan A gene *(PIG-A)* on chromosome Xp22.1 [1]. The *PIG-A* mutation is necessary but insufficient to explain PNH clone proliferation. The mechanism of the proliferative advantage of the PNH clone has not yet been clarified. At present, studies on the mechanism of PNH clone proliferation are mainly focused on protein-coding genes, while the function and clinical significance of non-coding RNAs (ncRNAs), and in particular long ncRNAs (LncRNAs), in PNH remain unknown. MALAT1 is one of the most extensively studied LncRNAs. It was found localized in the nucleus and expressed in a variety of tissues [2]. MALAT1 can regulate cell proliferation, differentiation, apoptosis, migration, and autophagy, among others [3]. MALAT1 has been demonstrated to be involved in many cancers, cardio-cerebrovascular disease, and hematological malignancies. However, knowledge of the action of MALAT1 in PNH is still lacking. The purpose of our study was to investigate the role of MALAT1 in PNH clone proliferation and to find a new therapeutic direction for PNH.

**Figure 1:**
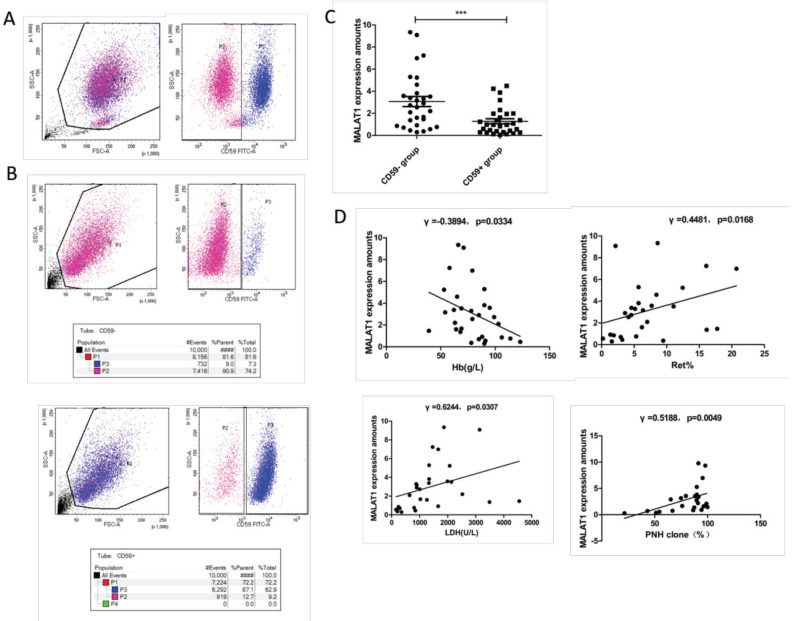
A) The cells of 30 PNH patients were sorted by flow cytometry to obtain CD59^-^ and CD59^+^ granulocytes and monocytes. Granulocytes and monocytes were selected as sorting objects in the first flow diagram, and CD59^-^ (P2) and CD59^+^ (P3) granulocytes and monocytes were selected in the second flow diagram by CD59 sorting. B) Sorting purity of the CD59^-^ and CD59^+^ granulocytes and monocytes. The sorting purity is about 90%. C) MALAT1 expression of the CD59^-^ and CD59^+^ cells in 30 PNH patients. D) Correlation analysis between MALAT1 expression and clinical data. The expression of MALAT1 was negatively correlated with the proportion of the level of Hb and positively correlated with the percentage of Ret, LDH levels, and the proportion of PNH clones.

A total of 30 PNH patients, including 22 PNH patients and 8 aplastic anemia (AA)-PNH patients, were enrolled in our study according to international PNH Study Group Criteria [4]. CD59^–^ and CD59^+^ granulocytes and monocytes were obtained by flow cytometry (Figures 1A and 1B). MALAT1 expressions were verified for these 30 PNH patients by quantitative real-time polymerase chain reaction (qRT-PCR). The clinical features of the PNH patients, primers of MALAT1, and methods of cell sorting and qRT-PCR are available from the authors as supplementary data. Correlations were analyzed with clinical indexes, including hemoglobin (Hb), white blood cell count (WBC), platelet count (PLT), reticulocytes (Ret), lactate dehydrogenase (LDH), total bilirubin (TBIL), and PNH clones.

As the results of qRT-PCR showed, MALAT1 (3.070±2.503) expressions in CD59^–^ cells were consistently higher than those in CD59^+^ cells (1.281±1.246, p=0.0004) among these 30 PNH patients (Figure 1C).

High expression of MALAT1 was negatively correlated with Hb level (r=-0.3894, p=0.0334) and positively correlated with the percentage of Ret (r=0.4481, p=0.0168), LDH levels (r=0.6244, p=0.0307), and CD59^–^ granulated and monocyte cell ratio (r=0.5188, p=0.0049) (Figure 1D).

The level of MALAT1 in PNH clone cells was found to be significantly increased and was correlated with clinical indicators of PNH. The molecular functions of MALAT1 include alternative splicing, transcriptional regulation, and competing endogenous RNA functions. MALAT1 was shown to bind alternative splicing factor SRSF1 in hepatocellular carcinoma development [5]. In another study, MALAT1 promoted the proliferation and imatinib resistance of chronic myeloid leukemia cells via the MALAT1/miR-328 axis [6]. MALAT1 downregulated miR-181a-5p through the Hippo-YAP signaling pathway, resulting in regulation of myeloma cell proliferation [7]. MALAT1 could also induce tolerogenic dendritic cells and immune tolerance in autoimmune diseases by regulating the miRNA-155/DC-SIGH/IL10 axis [8]. These results illustrate that MALAT1 plays an important role not only in malignant tumors but also in benign diseases.

The characteristics of clonal dynamics and selection forces of PNH clones are similar to those of tumors. Thus, we predict that MALAT1 may have an important function in contributing to proliferation advantages and restraining apoptosis in PNH progression. The mechanism remains to be further studied.

## Figures and Tables

**Supplemental Table 1:**
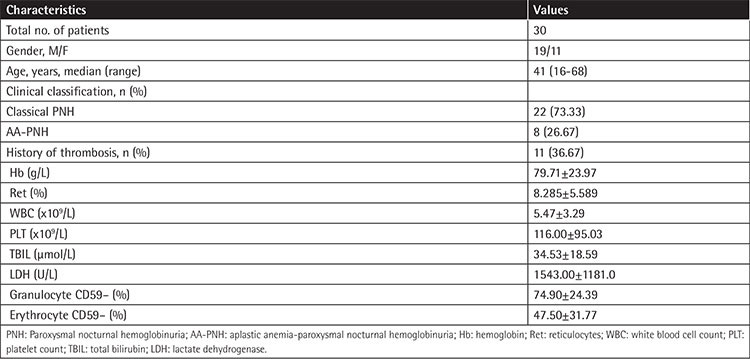
Clinical characteristics of 30 paroxysmal nocturnal hemoglobinuria patients.

**Supplemental Table 2:**
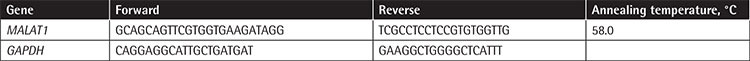
Gene primer sequences.
